# Cost-effectiveness of cemiplimab plus chemotherapy versus chemotherapy for the treatment of advanced non-small cell lung cancer

**DOI:** 10.3389/fonc.2023.1113374

**Published:** 2023-04-26

**Authors:** Xueyan Liang, Xiaoyu Chen, Huijuan Li, Yan Li

**Affiliations:** Department of Pharmacy, The People’s Hospital of Guangxi Zhuang Autonomous Region, Nanning, China

**Keywords:** aNSCLC, cemiplimab plus chemotherapy, chemotherapy, cost-effectiveness, partitioned survival model

## Abstract

**Background:**

In patients with advanced non-small cell lung cancer (aNSCLC), cemiplimab plus chemotherapy prolonged overall survival (OS) and progression-free survival (PFS) significantly compared to chemotherapy alone. The cost-effectiveness of these drugs is still uncertain. The aim of this study is to assess the cost-effectiveness of cemiplimab plus chemotherapy compared with chemotherapy for the treatment of aNSCLC from the third-party payer perspective in the United States.

**Materials and methods:**

The cost-effectiveness of cemiplimab with chemotherapy versus chemotherapy for the treatment of aNSCLC was evaluated using a partitioned survival model containing three mutually incompatible health states. The clinical characteristics and outcomes used in the model were gathered from EMPOWER-Lung 3 trial. We have conducted deterministic one-way sensitivity analysis and probabilistic sensitivity analysis in order to evaluate the robustness of the model. The primary outcomes considered were the costs, life-years, quality-adjusted life-years (QALYs), incremental cost-effectiveness ratio (ICER), incremental net health benefits (INHB), and incremental net monetary benefits (INMB).

**Results:**

Treatment of aNSCLC with cemiplimab plus chemotherapy increased efficacy by 0.237 QALYs and was associated with an increased total cost of $50,796 compared to chemotherapy alone, resulting in an ICER of $214,256/QALY gained. At a WTP threshold of $150,000/QALY, the INHB of cemiplimab plus chemotherapy was 0.203 QALYs and the INMB was $304,704 compared to chemotherapy alone. The probabilistic sensitivity analysis revealed that there was only a 0.04% chance that cemiplimab with chemotherapy would be cost-effective at a WTP threshold of $150,000/QALY. The performance of model was mainly determined by the price of cemiplimab, according to a one-way sensitivity analysis.

**Conclusions:**

From the third-party payer perspective, cemiplimab combined chemotherapy is unlikely to be a cost-effective option for the treatment of aNSCLC at the WTP threshold of $150,000/QALY in the United States.

## Introduction

Lung cancer is the common type of carcinoma and the leading cause of cancer death worldwide (1) with nearly 1.8 million people have died from lung cancer worldwide (2). The prognosis of lung cancer is poor since it is frequently diagnosed at an advanced stage. In the case of lung cancer, 85-90 percent are non-small cell lung cancers (NSCLCs) according to its histological categorization (3, 4). Approximately 50% of NSCLC patients progress to advanced or metastatic cancer (1, 5, 6). Similarly, a substantial number of individuals with local or locoregional illness progressed to recurrence or metastatic disease (7–9). In spite of the dismal prognosis for patients with distant metastatic disease, survival rates are reported to be approximately 5% at five years (1). New effective therapies for NSCLC are urgently required given the existing scenario.

Immune checkpoint inhibitors, which have steadily enhanced the therapy regimen for patients with NSCLC, have demonstrated their effectiveness in recent years (10). Lung cancer is an attractive context for current programmed cell death-1 (PD-1) and its ligand PD-L1 and Cytotoxic-T-lymphocyte-antigen-4 (CTLA-4) therapy due to its enhanced neo-antigen expression levels and ability to aid tumor cells in evading immune surveillance. The mainstay of systemic treatment for advanced non-small cell lung cancer (aNSCLC) is PD-1 and PD-L1 inhibitors in patients without therapeutically actionable tumor genomic aberrations, including epidermal growth factor receptor mutations, anaplastic lymphoma kinase translocations, or ROS proto-oncogene 1 (ROS1) fusions (10–12). Cemiplimab is a humanized recombinant monoclonal antibody that inhibits a high-affinity receptor (13, 14).

Cemiplimab was approved for marketing by the United States Food and Drug Administration (FDA) in September 2018 for the treatment of metastatic or locally advanced cutaneous squamous cell lung cancer due to it has potent antitumor activity and safety (15, 16). Therefore, cemiplimab seems to be a promising first-line immunotherapy option for treating aNSCLC.

Cemiplimab has significantly increased progression-free survival (PFS) and overall survival (OS) for the treatment of aNSCLC (17). For a significant number of aNSCLC patients, these exorbitant expenditures are untenable, and they are finally forced to forgo or delay treatment, impair their quality of life, or even declare bankruptcy (18–21). It is an extremely significant thing for doctors and decision-makers to analyze the cost-effectiveness of health choices in order to spend scarce health resources more judiciously and effectively. Due to the fact that both are licensed for the treatment of aNSCLC, clinicians and patients have difficulties deciding which is preferred, and a cost-effectiveness analysis is appropriate. This research aims to evaluate the cost-effectiveness of cemiplimab plus chemotherapy versus chemotherapy alone as the first-line treatment for aNSCLC.

## Materials and methods

### Patients and intervention

This study was conducted following the Consolidated Health Economic Evaluation Reporting Standards (CHEERS) standard (22). Based on the United States Department of Health and Human Services (45 CFR §46), this study did not use individual patient information, nor did it include human or animal research, so permission for ethical approval was not required from an institutional review board or ethics committee (23).

According to EMPOWER-Lung 3 trial (17), the inclusion criteria included men and women over the age of 18 (for Japanese patients, the age was 20); histologically or cytologically verified non-squamous or squamous NSCLC; Eastern Cooperative Oncology Group (ECOG) source ≤1; at least three months of expected life expectancy; and adequate organ and bone marrow function.

### Model structure

**Figure 1:**
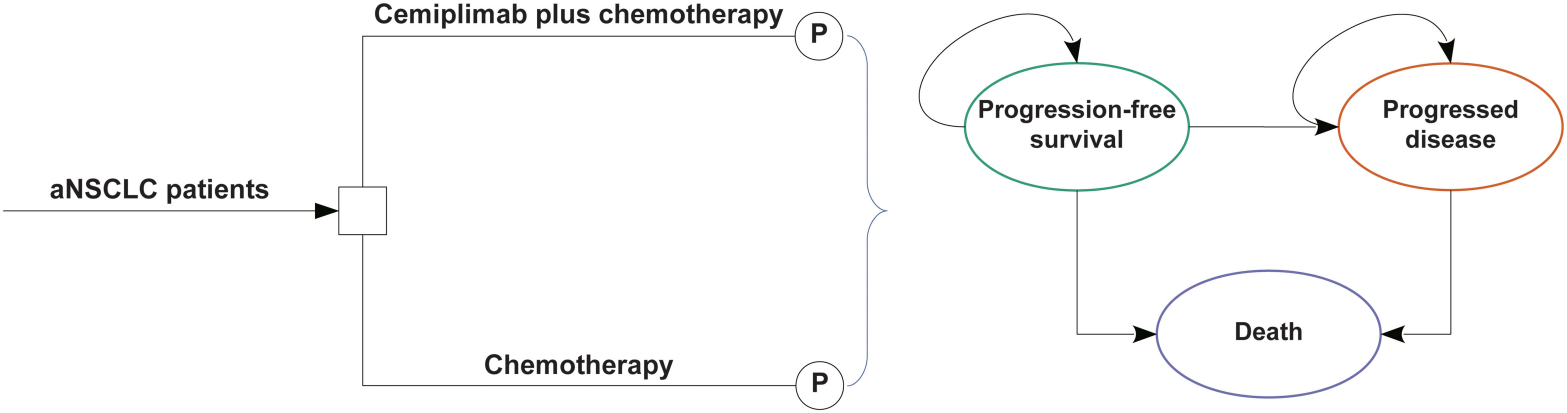
The partitioned survival model consisting of three discrete health states. aNSCLC, advanced non-small cell lung cancer; P indicates partitioned survival model.

We constructed a partitioned survival model including three mutually incompatible health states in this study: PFS, progressing disease (PD), and death (**Figure 1**). The area under the OS curve was assessed to determine the percentage of patients with OS, the area under the PFS curve was assessed to determine the proportion of patients with PFS, and based on the difference between OS and PFS curves, the proportion of patients with PD was determined.

The cycle length should be dictated by the natural history of disease and it should be the minimum interval over which the pathology or symptoms are expected to alter. Considering the progression of aNSCLC, the cycle length of the partitioned survival model was 1 week. The time horizon was 10 years that more than 98% of the cohort died. Throughout each cycle, the patients either maintained their current health status or advanced to the subsequent health level. The primary outcomes of this study were overall costs, incremental cost-effectiveness ratios (ICERs), quality-adjusted life-years (QALYs), life-years (LYs), incremental net health benefits (INHB), and incremental net monetary benefits (INMB). The threshold for willingness to pay (WTP) was set at $150,000/QALY (24). All cost and utility results were discounted annually by 3% (25, 26).

### Clinical data inputs

These OS and PFS survival curves are taken from the EMPOWER-Lung 3 study, which included cemiplimab in combination with chemotherapy and chemotherapy alone (17), and algorithm developed by Guyot et al. (27) was used to construct data beyond the follow-up period of the trial. In the EMPOWER-Lung 3 trial, which was conducted between June 17 2019 and September 30 2020, cemiplimab plus platinum-based chemotherapy was compared with patients who received platinum-based chemotherapy alone for the therapy of NSCLC (17).

We obtained Kaplan-Meier survival curves from the EMPOWER-Lung 3 trial by GetData Graph Digitizer version 2.26 (28), which allows time-to-survival data points to be extracted. In order to fit this data, parametric survival models were used: Weibull, log-normal, log-logistic, exponential, generalized gamma, and Gompertz. The optimal survival model was chosen based on Akaike information criteria and Bayesian information criterion with the lowest scores. The survival fit model results of cemiplimab plus chemotherapy and chemotherapy alone were displayed in **Table 1**, and **Supplementary Table 1** displays the results of fit in detail. The proportions of patients with PFS and OS were calculated by using the selected survival distribution. It was found that the survival events and survival durations of virtual patients were comparable to the actual number of patients at risk, which was indicative of a close replication of the survival curves. Further details of the model fitting are presented in **Supplementary Figure 1**.

**Table 1:** Key model inputs.

Parameter	Value (95% CI)	Distribution	Source
Weibull OS survival model of cemiplimab plus chemotherapy^a^	γ = 1.1539 λ = 0.0044	Weibull	Gogishvili et al., 2022 (17)
Weibull PFS survival model of cemiplimab plus chemotherapy^a^	γ = 1.2330 λ = 0.0076	Weibull	Gogishvili et al., 2022 (17)
Weibull OS survival model of chemotherapy^a^	γ = 1.2353 λ = 0.0047	Weibull	Gogishvili et al., 2022 (17)
Weibull PFS survival model of chemotherapy^a^	γ = 1.3439 λ = 0.0095	Weibull	Gogishvili et al., 2022 (17)
Body surface area, m^2^	1.86 (1.40 to 2.23)	Gamma	Pei et al., 2021 (23)
Body weight, kg	70 (50 to 91)	Gamma	Pei et al., 2021 (23)
Drug costs per 1 mg			
Price of cemiplimab	27.54 (20.66 to 34.43)	Gamma	CMS
Price of pemetrexed	7.49 (5.62 to 9.36)	Gamma	CMS
Price of paclitaxel	0.13 (0.1 to 0.16)	Gamma	CMS
Price of carboplatin	0.05 (0.04 to 0.07)	Gamma	CMS
Price of cisplatin	0.18 (0.13 to 0.22)	Gamma	CMS
Cost of terminal care per patient^d^	16442 (12331 to 20552)	Gamma	Insinga et al., 2019 (29)
Administration cost			
First hour	158.7 (130.01 to 206.05)	Gamma	CPT code 96413
Additional hour	33.6 (28.35 to 42.31)	Gamma	CPT code 96415
Cost of managing AEs (grade ≥ 3)^c^			
Cemiplimab plus chemotherapy	2844 (2133 to 3377)	Gamma	Konidaris et al., 2021 (30); Wong et al., 2018 (31); Jeong et al., 2021 (32)
Chemotherapy	936 (702 to 1111.5)	Gamma	Konidaris et al., 2021 (30)
Disease costs per cycle			
Stable disease	464.85 (348.64 to 581.06)	Gamma	Insinga et al., 2019 (29)
Progressed disease	1075.49 (806.62 to 1344.36)	Gamma	Insinga et al., 2019 (29)
Health utilities			
Disease status utility per year			
Stable disease	0.754 (0.407 to 0.970)	Beta	Nafees et al., 2017 (33) Nafees et al., 2017 (33)
Disease progression	0.180 (0.115 to 0.367)	Beta	Nafees et al., 2017 (33) Nafees et al., 2008 (34)
Death	0	NA	
Disutility due to AEs^d^			
Cemiplimab plus chemotherapy	0.044 (0.033 to 0.052)	Beta	Nafees et al., 2017 (33); Freeman et al., 2015 (35); Jeong et al., 2021 (32)
Chemotherapy	0.015 (0.011 to 0.018)	Beta	Nafees et al., 2017 (33); Freeman et al., 2015 (35); Jeong et al., 2021 (32)

AE, adverse event; NA, not applicable; OS, overall survival; PD, progressed disease; PFS, progression-free survival.

a Only expected values are presented for these survival model parameters.

b Overall total cost per patient regardless of treatment duration.

c Calculated as the average cost of toxic effects using weighted frequencies of grade ≥ 3 treatment related AEs for each treatment arm in the EMPOWER-Lung 3 trial. Costs of individual toxic effects were derived from the literature and include all care required to manage each toxic effect. References for individual toxic effect costs are summarized in **Supplementary Table 2**.

d Calculated as the average disutility of toxic effects using weighted frequencies of grade ≥ 3 treatment-related AEs for each treatment arm in the EMPOWER-Lung 3 trial. Disutilities of individual toxic effects were derived from the literature. References for individual toxic effect disutilities are summarized in **Supplementary Table 2**.

### Cost and utility inputs

We evaluated the costs that included direct medical charges. Direct medical costs consist of prescription costs, patient health-related costs, adverse event (AE) management costs, and costs related to terminal care (**Table 1**). According to medical-care inflation from Tom’s Inflation Calculator (36), all costs have been converted to 2021 United States dollars (**Table 1**). According to the results of the EMPOWER-Lung 3 study, patients in cemiplimab plus chemotherapy group received 350 mg of cemiplimab every three weeks in addition to chemotherapy. Researchers used paclitaxel plus carboplatin, paclitaxel plus cisplatin, pemetrexed plus carboplatin, or pemetrexed plus cisplatin as chemotherapy treatments. There was a maximum duration of treatment of 108 weeks, or until the disease progressed or toxicity became intolerable. To assess direct drug prices, the Centers for Medicare and Medicaid Services (CMS) was used to offer the 2021 average retail price for drugs (37). We estimated the chemotherapy dosage based on the assumption that a typical patient has a body surface area of 1.86 m^2^ and a weight of 70 kg (23). Monitoring costs for patients in the PFS and PD stages were $465 and $1,075 per cycle, respectively (29). The cost of terminal care is $16441.83 per aNSCLC patient (29). This study calculated the costs associated with addressing grade ≥ 3 AEs based on the literature (**Supplementary Table 2**) (30–32).

There was an associated health value for aNSCLC health status ranging from 0 (death) to 1 (perfect health). The utilities of PFS and PD states for aNSCLC were 0.754 and 0.18, respectively (33, 34). This research assessed the disutility based on grade ≥ 3 AEs and the literature-derived estimate of disutility attributable to treatment-emergent AEs grade ≥3 (**Supplementary Table 2**) (32, 33, 35).

### Base-case analysis

ICER was represented as the cost per extra QALY gained between cemiplimab plus chemotherapy and chemotherapy alone. When the incremental cost-effectiveness ratio (ICER) is below a certain WTP threshold, cost-effectiveness is recommended (24). The INHB and INMB were presented as the following formulas: $\begin{array}{l} \text{INHB}\left(\text{λ}\right)=\left(\text{μ}E_{\text{Cemiplimab plus chemotherapy}}\;-\text{ μ}E_{Chemotherapy}\right)\\ -\frac{\left(\text{μ}C_{\text{Cemiplimab plus chemotherapy}}\;-\text{ μ}C_{Chemotherapy}\right)}{\text{λ}}=\;\Delta E-\;\frac{\Delta C}{\lambda } \end{array}$ and $\begin{array}{l} \text{INMB}\left(\text{λ}\right)=\left(\text{μ}E_{\text{Cemiplimab plus chemotherapy}}\;-\text{ μ}E_{Chemotherapy}\right)\\ \;\times \;\lambda -\left(\text{μ}C_{\text{Cemiplimab plus chemotherapy}}\;-\text{ μ}C_{Chemotherapy}\right)=\;\Delta E\;\times \;\lambda \;–\;\Delta C \end{array}$ , where μC and μE were the cost and utility of cemiplimab plus chemotherapy or chemotherapy, respectively, and λ was the WTP threshold (38, 39).

### Sensitivity analysis and probabilistic sensitivity analysis

To determine the robustness of the model outputs, we conducted one-way sensitivity analysis and probabilistic sensitivity studies in this study. One-way sensitivity analysis were conducted based on various variables, such as costs and utilities. In order to estimate the uncertainty of each variable, we either used the 95% confidence intervals provided by the literature or approximated it by assuming a 25% deviation from the baseline value (**Table 1**). In order to evaluate the uncertainty of the model, we used Monte Carlo simulation with 10,000 iterations to perform a probabilistic sensitivity analysis. The cost parameters were assigned a gamma distribution, the hazard ratios (HRs) were assigned a log-normal distribution, and the probability, percentage, and utility parameters were assigned a beta distribution. A cost-effectiveness acceptability curve was constructed to determine whether cemiplimab plus chemotherapy or chemotherapy could benefit QALY gains at different WTP levels.

### Subgroup analysis

Subgroup analysis was performed to investigate the uncertainty of the model resulting from the various patient characteristics. Variables, such as age, sex, race, histology, PD-L1 expression level, ECOG, area, brain metastases, cancer stage, and smoking history were used while performing subgroup analysis for the various subgroups produced by EMPOWER-Lung 3 (17). In this study, the hesim and heemod packages in R, version 4.0.5, 2021 (R Foundation for Statistical Computing), were used to performed statistical analysis.

## Results

### Base-case analysis

Compared to chemotherapy, cemiplimab plus chemotherapy delivered an extra 0.237 QALYs at an additional cost of $50,796, yielding an ICER of $214,256/QALY. An INHB of 0.203 and an INMB of $304,704 were found at a WTP threshold of $150,000/QALY (**Table 2**).

**Table 2:** Summary of cost and outcome results in the base-case analysis.

Factor	Cemiplimab plus chemotherapy	Chemotherapy	Incremental change
Cost, $			
Druga	49,600	11,218	38,382
Nondrugb	81,589	69,175	12,414
Overall	131,189	80,393	50,796
Life-years			
Progression-free	0.866	0.563	0.303
Overall	1.821	1.385	0.436
**QALYs**	0.796	0.559	0.237
ICERs, $			
Per life-year	NA	NA	116,673
Per QALY	NA	NA	214,256
INHB, QALY, at threshold 150,000a	NA	NA	0.203
INMB, $, at threshold 150,000a	NA	NA	304,704

ICER, incremental cost-effectiveness ratio; INHB, incremental net health benefit; INMB, incremental net monetary benefit; NA, not applicable; QALYs, quality-adjusted life years.

a Compared with chemotherapy.

b Nondrug cost includes the costs of adverse event management, subsequent best supportive care per patient, and follow-up care covering physician monitors, drug administration, and terminal care.

### Sensitivity analysis and probabilistic sensitivity analysis

As a result of one-way sensitivity analysis, the primary driver of the model result is the cost of cemiplimab (**Supplementary Figure 2**), as it had the greatest impact on ICER. Model results were robust to the uncertainty of other model variables, including costs and disutility associated with AE risk.

These key factors were assessed for their relevance to ICER of cemiplimab plus chemotherapy compared to chemotherapy alone. When the WTP threshold was set at $150,000/QALY and the cost of cemiplimab was less than $15.91 per mg, cemiplimab plus chemotherapy could be considered cost-effective (**Supplementary Figure 3**).

**Figure 2:**
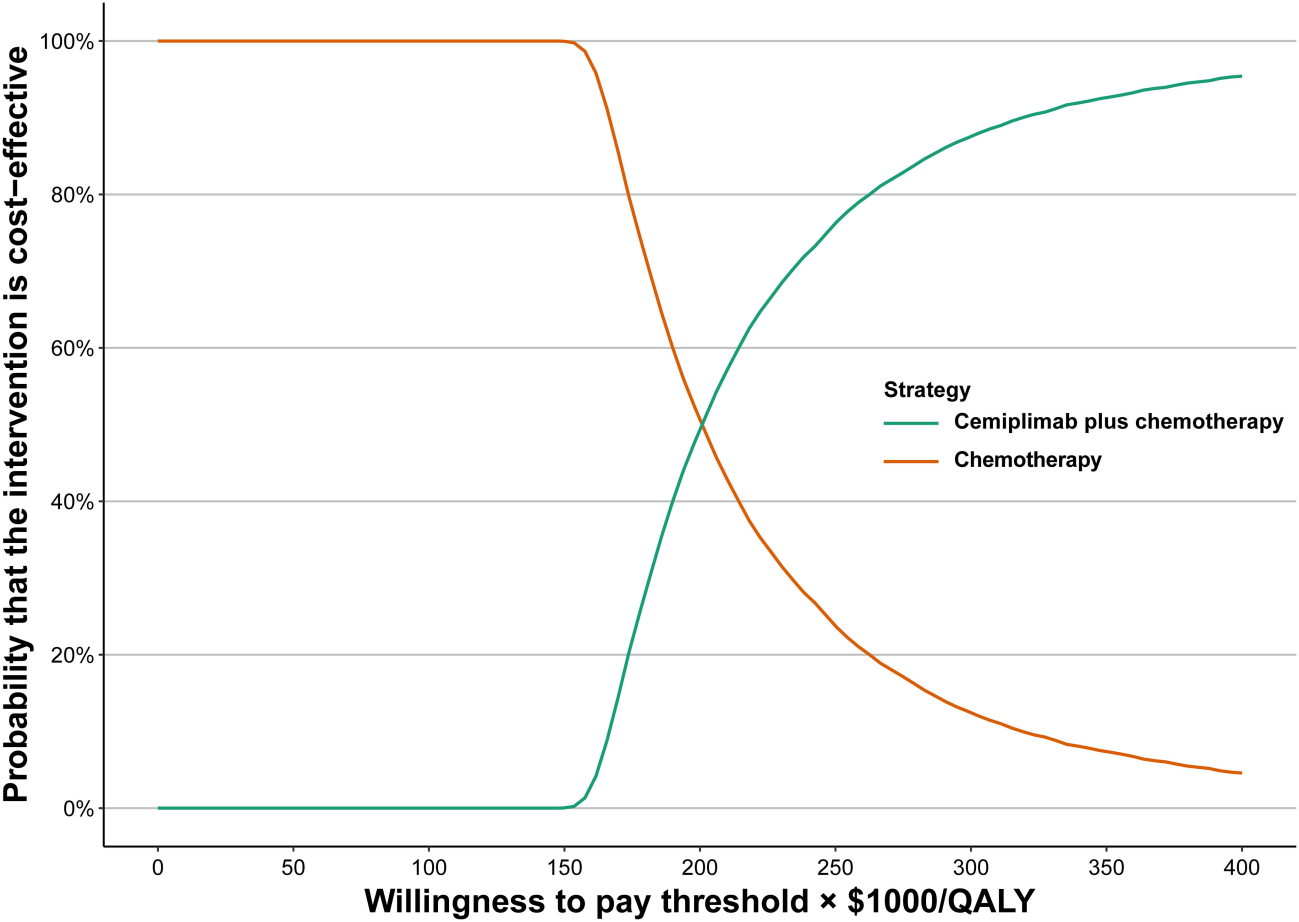
Acceptability curves of cost-effectiveness for cemiplimab plus chemotherapy versus chemotherapy.

Based on the cost-effectiveness acceptability curves and ICER scatterplot, cemiplimab plus chemotherapy has a 0.04% chance to be considered as cost-effective when compared with chemotherapy alone when the WTP threshold is set at $150,000 (**Figure 2**; **Supplementary Figure 4**).

### Subgroup analysis

The subgroup analysis, which were performed based on varying the HRs for OS, found that cemiplimab plus chemotherapy was related to a higher than 50% likelihood of being cost-effective at the threshold of $150,000/QALY in the following subgroups (**Table 3**): female patients, patients with PD-L1 expression ≥50%, patients with an ECOG score of 1, European patients, and never smokers. The INHBs in subgroups ranged from -0.24 for patients with brain metastasis to 0.02 for patients from Europe.

**Table 3:** Summary of subgroup analysis obtained by varying the hazard ratios (HRs) for overall survival.

Subgroup	Unstratified HR for OS (95% CI)	Change in cost, $^a^	Change in QALY^a^	ICER, $/QALY	Cost-effectiveness probability of cemiplimab plus chemotherapy, %, at WTP of $150,000/QALY	INHB at WTP of $150,000
Age group						
< 65 years	0.57 (0.40–0.81)	67,324	0.292	230,237	0	-0.16
≥ 65 years	0.88 (0.56–1.37)	36,614	0.190	193,110	26.19	-0.05
Sex						
Male	0.55 (0.41–0.74)	68,741	0.297	231,329	0	-0.16
Female	2.11 (0.89–5.03)	23,927	0.147	162,624	57.3	-0.01
Race						
White	0.67 (0.50–0.89)	54,966	0.251	218,952	0	-0.12
Non-white	0.79 (0.31–2.02)	43,495	0.213	204,549	5.23	-0.08
Histology						
Squamous	0.56 (0.37–0.84)	68,741	0.297	231,329	0	-0.16
Non-squamous	0.79 (0.54–1.14)	43,495	0.213	204,549	5.37	-0.08
PD-L1 level						
< 1%	1.01 (0.63–1.60)	28,541	0.163	175,555	47.14	-0.03
1-49%	0.52 (0.32–0.83)	74,796	0.317	235,632	0	-0.18
≥ 50%	0.61 (0.37–1.02)	56,970	0.384	148,188	76.05	0.00
ECOG PS						
0	0.55 (0.20–1.49)	70,194	0.302	232,415	0	-0.17
1	0.69 (0.52–0.92)	49,702	0.360	138,025	69.45	0.03
Region						
Europe	0.67 (0.50–0.90)	51,498	0.366	140,662	71.82	0.02
Asia	0.72 (0.27–1.88)	49,812	0.234	213,066	0.17	-0.10
Brain metastasis						
Yes	0.42 (0.14–1.26)	93,247	0.379	245,907	0	-0.24
No	0.68 (0.51–0.90)	53,887	0.247	217,788	0.05	-0.11
Cancer stage						
Locally advanced	0.54 (0.25–1.15)	71,687	0.307	233,494	0	-0.17
Metastatic	0.69 (0.51–0.93)	52,833	0.244	216,617	0.02	-0.11
Smoking						
Smokers	0.61 (0.46–0.82)	62,011	0.275	225,801	0	-0.14
Never smokers	1.28 (0.53–3.08)	26,406	0.155	169,891	52.55	-0.02

ECOG PS: Eastern Cooperative Oncology Group performance status; HR, hazard ratio; ICER, incremental cost-effectiveness ratio; INHB, incremental net health benefits; OS, overall survival; PD-L1, programmed cell death ligand 1; QALY, quality-adjusted life-year; WTP, willingness to pay.

a HR for OS represents the HR of cemiplimab plus chemotherapy vs. chemotherapy for OS; change in cost and change in QALYs represent the results of cemiplimab plus chemotherapy minus chemotherapy.

In subgroup analysis conducted by altering the HRs for PFS, cemiplimab with chemotherapy was associated with a likelihood of cost-effectiveness greater than 50% in the following subgroups (**Table 4**): patients with PD-L1 expression 1-49% and ≥50%, patients with an ECOG score of 0, and patients with locally advanced disease.

**Table 4:** Summary of subgroup analysis obtained by varying the hazard ratios (HRs) for progression-free survival.

Subgroup	Unstratified HR for PFS (95% CI)	Change in cost, $^a^	Change in QALY^a^	ICER, $/QALY	Cost-effectiveness probability of cemiplimab plus chemotherapy, %, at WTP of $150,000 per QALY	INHB at WTP of $150,000
Age group						
< 65 years	0.53 (0.39–0.71)	49,933	0.255	195,473	8.08	-0.08
≥ 65 years	0.56 (0.39–0.81)	50,826	0.236	214,948	0.06	-0.10
Sex						
Male	0.48 (0.37–0.61)	48,067	0.294	163,288	47.25	-0.03
Female	0.90 (0.50–1.62)	54,051	0.165	328,427	0	-0.20
Race						
White	0.54 (0.43–0.69)	50,189	0.250	200,728	3.41	-0.08
Non-white	0.58 (0.28–1.20)	51,361	0.225	228,376	0	-0.12
Histology						
Squamous	0.56 (0.40–0.79)	50,855	0.236	215,641	0.03	-0.10
Non-squamous	0.53 (0.39–0.73)	49,868	0.257	194,172	9.82	-0.08
PD-L1 level						
< 1%	0.76 (0.51–1.15)	54,901	0.144	380,177	0	-0.22
1–49%	0.47 (0.33–0.68)	47,662	0.303	157,460	53.31	-0.02
≥ 50%	0.47 (0.31–0.72)	47,744	0.301	158,617	52.43	-0.02
ECOG PS						
0	0.20 (0.09–0.43)	41,810	0.420	99,654	86.92	0.14
1	0.60 (0.47–0.76)	51,888	0.213	243,132	0	-0.13
Region						
Europe	0.55 (0.43–0.70)	50,498	0.243	207,421	0.66	-0.09
Asia	0.52 (0.25–1.10)	49,535	0.264	187,749	18.25	-0.07
Brain metastasis						
Yes	0.53 (0.22–1.31)	49,901	0.256	194,822	8.88	-0.08
No	0.54 (0.43–0.69)	50,221	0.249	201,391	2.83	-0.09
Cancer stage						
Locally advanced	0.34 (0.19–0.62)	43,109	0.394	109,399	82.55	0.11
Metastatic	0.59 (0.46–0.75)	51,629	0.219	235,671	0	-0.13
Smoking						
Smokers	0.53 (0.42–0.68)	49,852	0.257	193,848	10.19	-0.08
Never smokers	0.65 (0.34–1.22)	53,058	0.187	283,153	0	-0.17

ECOG PS: Eastern Cooperative Oncology Group performance status; HR, hazard ratio; ICER, incremental cost-effectiveness ratio; INHB, incremental net health benefits; PFS, progression-free survival; PD-L1, programmed cell death ligand 1; QALY, quality-adjusted life-year; WTP, willingness to pay.

a HR for PFS represents the HR of cemiplimab plus chemotherapy vs. chemotherapy for PFS; change in cost and change in QALYs represent the results of cemiplimab plus chemotherapy minus chemotherapy.

## Discussion

In this study, we conducted a cost-effectiveness analysis of combining cemiplimab with chemotherapy versus chemotherapy for the treatment of aNSCLC. The analysis suggested that compared with chemotherapy alone, cemiplimab plus chemotherapy was related to an incremental survival of 0.237 QALYs and an extra cost of $50,796 per patient, and the ICER of cemiplimab plus chemotherapy was estimated to be $214,256/QALY. At a WTP threshold of $150,000/QALY, cemiplimab plus chemotherapy would not be considered cost-effective. Based on one-way sensitivity analysis, cost of cemiplimab was found to be the key factor on the ICER, revealing that the decision between cemiplimab plus chemotherapy and chemotherapy alone may consider cemiplimab prices. There has been a comprehensive assessment of the sensitivity of this model using both one-way sensitivity analysis and probabilistic sensitivity analysis. At a WTP threshold of $150,000/QALY, the cost-effectiveness acceptability curves showed that cemiplimab plus chemotherapy had a 0.04% chance of being considered as cost-effective.

This is the first study to our knowledge to evaluate the cost-effectiveness of cemiplimab plus chemotherapy for the therapy of aNSCLC. In this cost-effectiveness analysis, cemiplimab plus chemotherapy was found to be less cost-effective than chemotherapy alone in treating patients with aNSCLC. Prior studies assessed the cost-effectiveness of cemiplimab monotherapy vs platinum-based chemotherapy for patients with aNSCLC with high PD-L1 expression, generating an ICER of $40,390/QALY in patients with aNSCLC with high PD-L1 expression (40). In a recently published trial comparing cemiplimab and pembrolizumab for patients with aNSCLC and high PD-L1 expression, pembrolizumab resulted in a societal ICER of $114,246/QALY from a societal perspective in the United States compared to cemiplimab alone (41).

It is important to highlight the advantages of this study. First, this is the first research that, to our knowledge, used a partitioned survival model to compare the cost-effective of cemiplimab plus chemotherapy versus chemotherapy for the treatment of aNSCLC. Second, cemiplimab plus chemotherapy was unlikely to be considered a cost-effectiveness alternative versus chemotherapy for the treatment of patients with aNSCLC. Third, the economic results of this study may be helpful to patients and physicians when customizing treatment choices.

There are some limitations to this study that should be noted. First, the reported Kaplan-Meier survival curves of OS and PFS data were fitted using parametric distributions in order to account for health outcomes that occurred beyond the follow-up period of the EMPOWER-Lung 3 study. This may have led to uncertainty in the predictions of the model. The findings of the sensitivity analysis show that this conclusion is typically robust, suggesting that this constraint may not be a significant factor. Second, we hypothesized that the risk of AEs and the proportion of patients managed for AEs were the same in the subgroup as in the therapy groups. Third, the robustness of the model was estimated by evaluating the structure of model and assumptions, and variable sources. Our sensitivity analysis included an evaluation of uncertainties. Nevertheless, cemiplimab is a relatively new therapy for patients with aNSCLC, long-term survival statistics were not available, and the results and conclusion of this study need to be further evaluated and examined. Fourth, the face validation of the model was judged by experts, including model structure, assumptions, data sources, analyses, and results. All the uncertainties suggested by the experts were included in the sensitivity analysis. We assumed a variance of 25% in the baseline values of variables which was not provided the range of confidence intervals for the values. The assumption method is frequently employed in economic evaluations, although it is possible for some variables to have inaccurate intervals. Fifth, cemiplimab plus chemotherapy is relatively new for the treatment of patients with aNSCLC, long-term observational data were unavailable to externally validate the extrapolation of the models, and long-term PFS and OS needed to be projected based on fitting curves to the observed trial data. Although numerous survival curve options have been included in the model for sensitivity analysis purposes, uncertainty still remains about survival extrapolations beyond the trials; however, our results did not appear to be particularly sensitive to the extrapolated parameter functions. Finally, due to regional disparities in cost inputs and payment capabilities, the findings of this study may not be applicable to other nations.

## Conclusions

From a third-party payer perspective in the United States, cemiplimab plus chemotherapy is unlikely to be a cost-effective first-line treatment option for patients with aNSCLC exceeding a WTP threshold of $150,000/QALY compared with chemotherapy. When deciding the cost-effectiveness between cemiplimab plus chemotherapy and chemotherapy for the therapy of aNSCLC, it was also discovered that the WTP threshold should be taken into account. Although immunotherapy is a promising field of cancer treatment, its high costs must be considered in order to give the best patient care.

## Data availability statement

The original contributions presented in the study are included in the article/**Supplementary Material**. Further inquiries can be directed to the corresponding author.

## Ethics statement

Ethical review and approval was not required for the study on human participants in accordance with the local legislation and institutional requirements. Written informed consent for participation was not required for this study in accordance with the national legislation and the institutional requirements.

## Author contributions

XL was involved in the conception and design of the study, constructed the model, conducted a literature search and gathered data, analyzed the data, and drafted the paper. XC conceived and designed the study, developed the model, performed the literature search and data acquisition, analyzed the data, and revised the manuscript. HL contributed to the interpretation of the results and to the revision of the manuscript for intellectual content that was of significance. YL designed and conceptualized the study, obtained data, revised the manuscript, provided technical and material support, and approved the final version. All authors contributed to the article and approved the submitted version.
