# Supplementary material for: Cost-effectiveness of cemiplimab plus chemotherapy versus chemotherapy for the treatment of advanced non-small cell lung cancer
Source: Front Oncol. 2023 Apr 26;13:1113374. doi: 10.3389/fonc.2023.1113374 (PMC10171429; doi:10.3389/fonc.2023.1113374)
Supplement: Supplementary file 1 [file DataSheet_1.docx]

**Supplementary Content**

**Supplementary Figure 1.** Model Fitting Analysis

**Supplementary Figure 2.** Tornado Diagram of One-Way Sensitivity Analysis of Cemiplimab Plus Chemotherapy Versus Chemotherapy.

**Supplementary Figure 3.** One-Way Sensitivity Analysis Result of ICER When Varying Cost of Cemiplimab in aNSCLC Partients.

**Supplementary Figure 4.** Results of Monte Carlo Probabilistic Sensitivity Analysis Showing Incremental Cost-effectiveness of Cemiplimab Plus Chemotherapy Versus Chemotherapy.

**Supplementary Table 1.** Estimated Parameters and AIC and BIC Values from Each Survival Model.

**Supplementary Table 2.** Associated Costs and Disutility of Grade ≥ 3 Treatment-Related Adverse Events

**Supplementary Figure 1.** Model Fitting Analysis

To obtain the best model fit, the following investigations were carried out using cemiplimab plus chemotherapy or chemotherapy as the model fit baseline, respectively. Based on AIC and BIC (Supplementary Table 1). CPS, combined positive score.

(A) Model-fitted versus original K-M curves for cemiplimab plus chemotherapy aNSCLC patients, Weibull model were used to fit the OS and PFS K-M of cemiplimab plus chemotherapy.


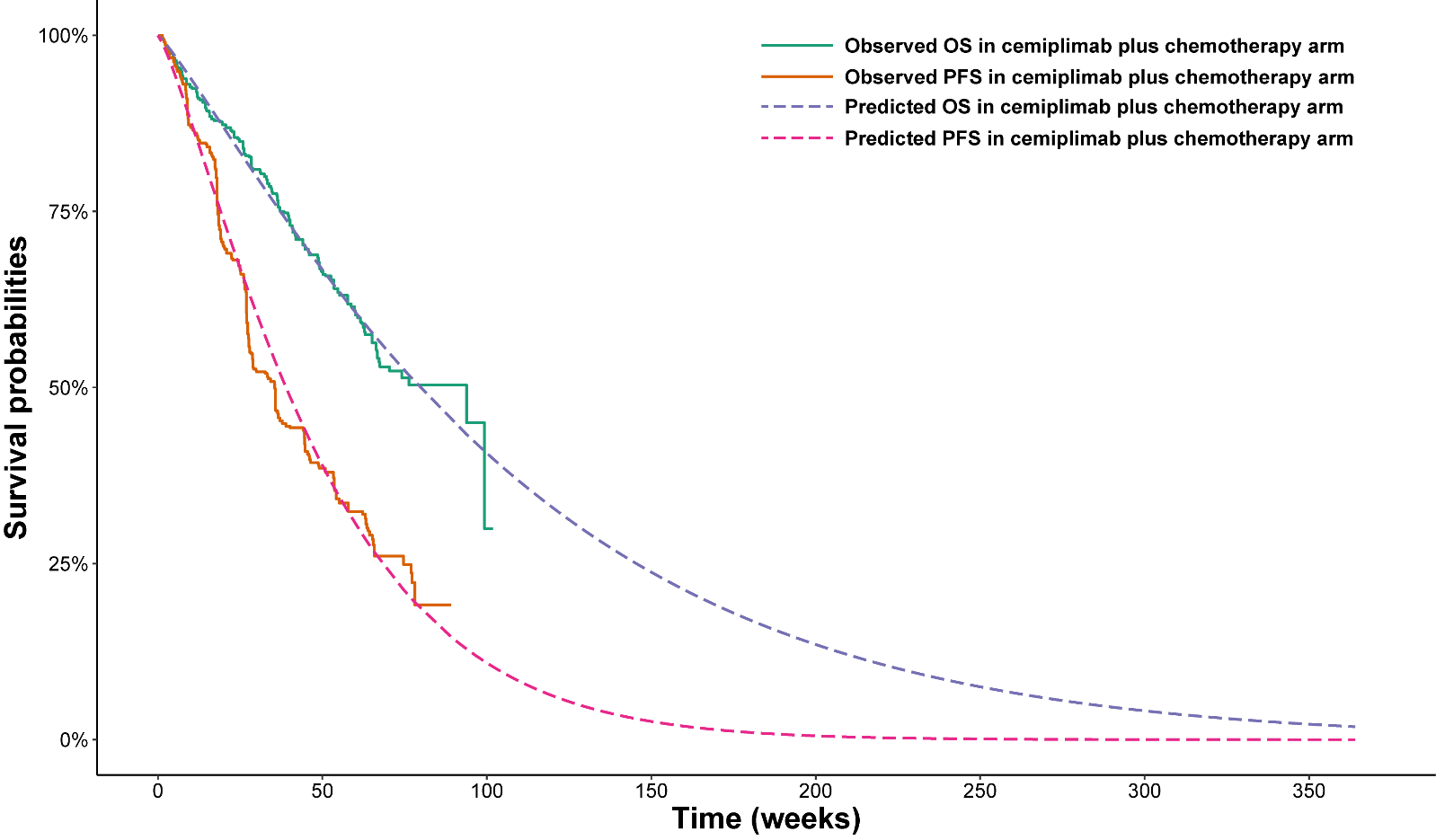


(B) Model-fitted versus original K-M curves for chemotherapy in aNSCLC patients, Weibull model was used to fit the OS and PFS K-M of chemotherapy.


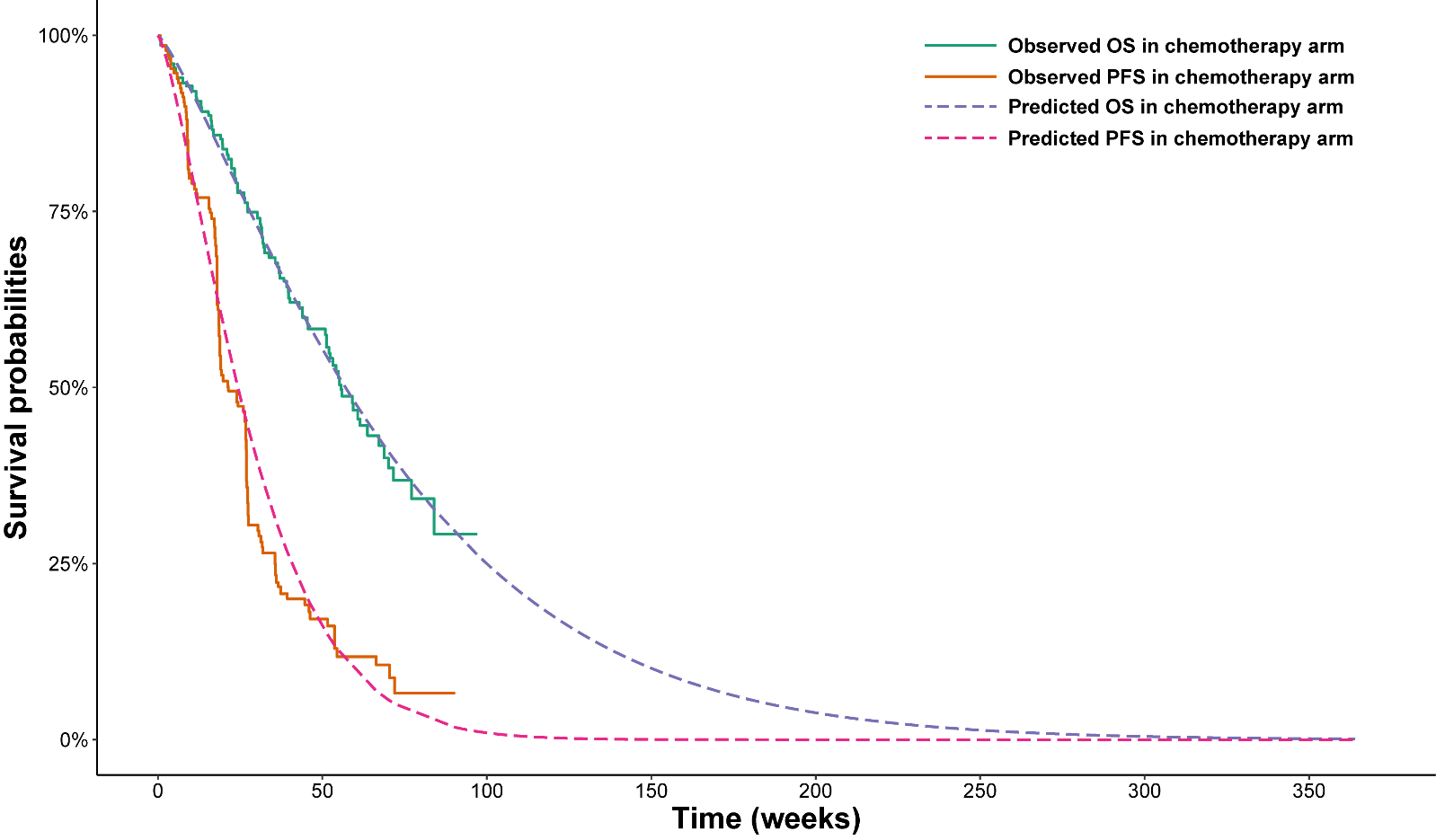


**Supplementary Figure 2.** Tornado Diagram of One-Way Sensitivity Analysis of Cemiplimab Plus Chemotherapy Versus Chemotherapy.


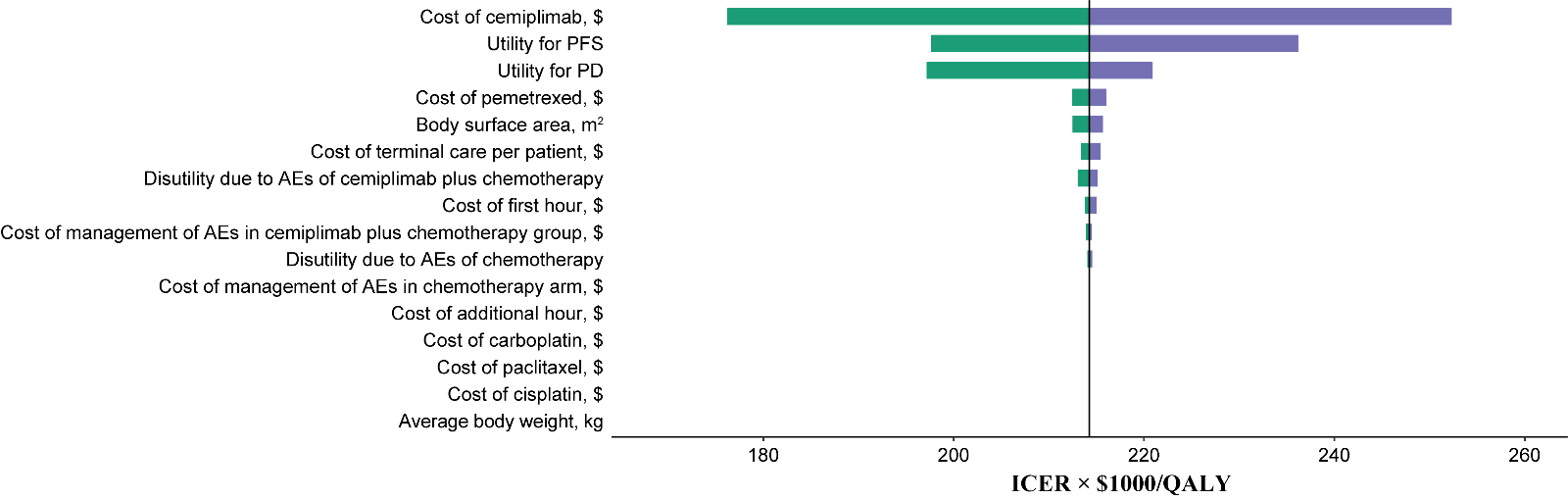


**Supplementary Figure 3.** One-Way Sensitivity Analysis Result of ICER When Varying Cost of Cemiplimab in aNSCLC Partients. Graphs represent the ICERs of cemiplimab plus chemotherapy compared with chemotherapy.


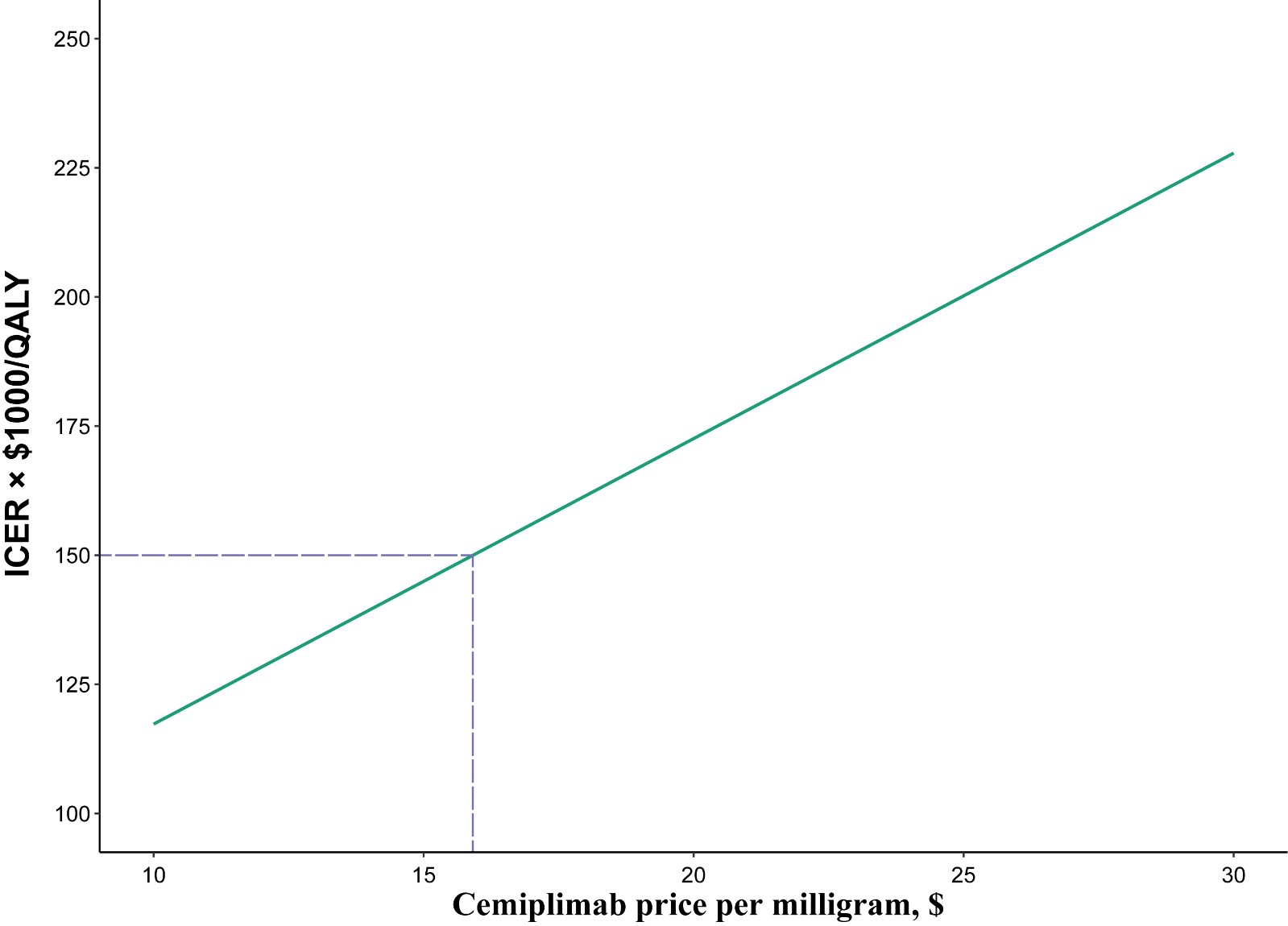


**Supplementary Figure 4.** Results of Monte Carlo Probabilistic Sensitivity Analysis Showing Incremental Cost-effectiveness of Cemiplimab Plus Chemotherapy Versus Chemotherapy.


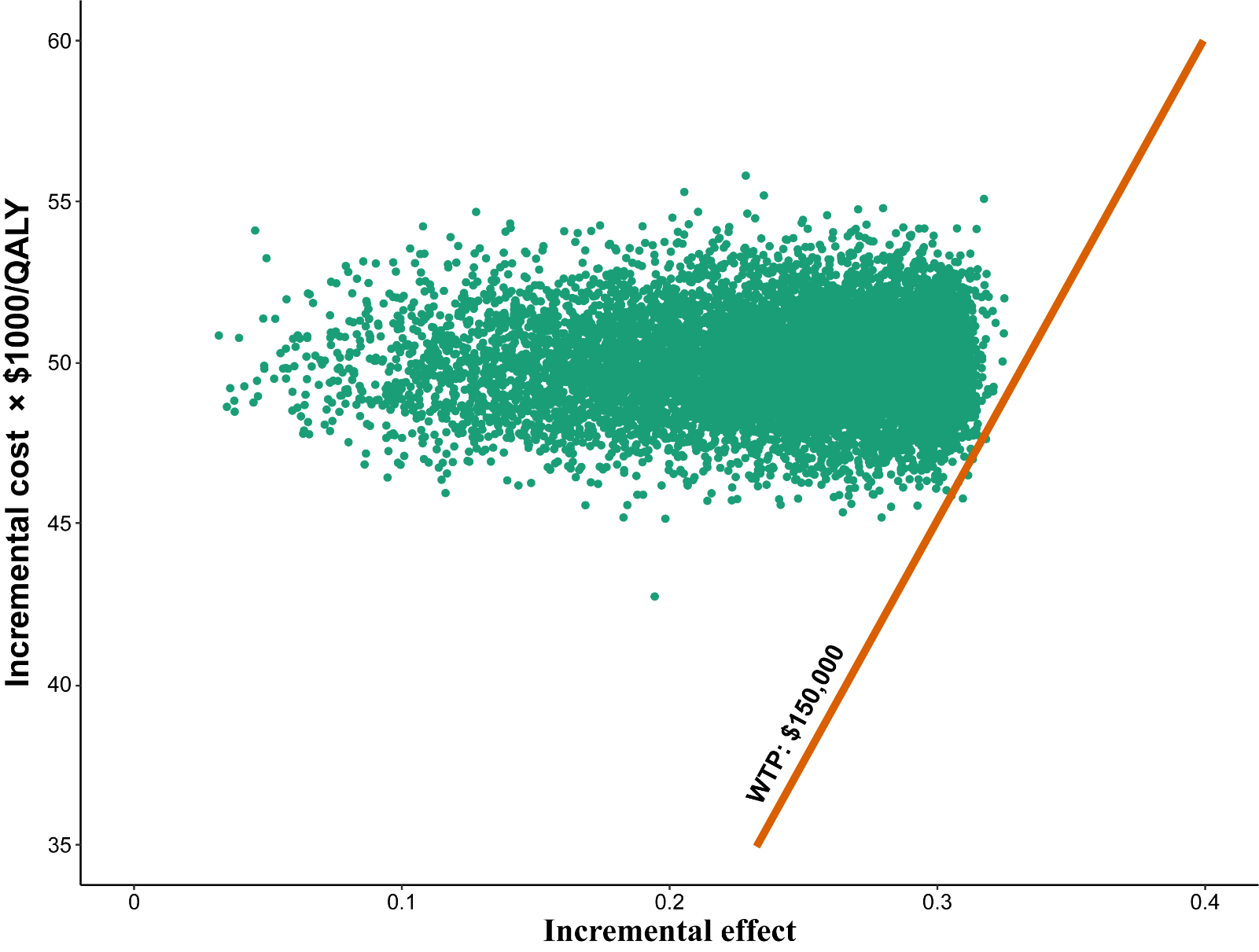


**Supplementary Table 1.** Estimated Parameters and AIC and BIC Values from Each Survival Model.

| **Strategies** | **Distributions** | **Parameters** | **est** | **se** | **L95%** | **U95%** | **AIC** | **BIC** |
| --- | --- | --- | --- | --- | --- | --- | --- | --- |
| **Results of OS** | | | | | | | | |
| **Cemiplimab plus chemotherapy** | Exponential | rate | 0.0084 | 0.0007 | 0.0071 | 0.0099 | 1586.992 | 1590.735 |
|  | Weibull | shape | 1.1539 | 0.0889 | 0.9922 | 1.3420 | **1585.728** | **1593.215** |
|  |  | scale | 0.0044 | 0.0017 | 0.0021 | 0.0093 |  |  |
|  | Gamma | shape | 1.1977 | 0.1199 | 0.9843 | 1.4574 | 1585.87 | 1593.356 |
|  |  | rate | 0.0111 | 0.0019 | 0.0079 | 0.0154 |  |  |
|  | Lognormal | meanlog | 4.4605 | 0.1065 | 4.2518 | 4.6692 | 1593.367 | 1600.853 |
|  |  | sdlog | 1.4176 | 0.0938 | 1.2451 | 1.6140 |  |  |
|  | Gompertz | shape | 0.0063 | 0.0036 | -0.0007 | 0.0134 | 1585.977 | 1593.463 |
|  |  | rate | 0.0067 | 0.0011 | 0.0049 | 0.0091 |  |  |
|  | Log-logistic | shape | 1.3221 | 0.0997 | 1.1404 | 1.5326 | 1588.084 | 1595.57 |
|  |  | scale | 82.5442 | 7.4291 | 69.1953 | 98.4683 |  |  |
|  | Generalized gamma | mu | 4.7194 | 0.1147 | 4.4946 | 4.9443 | 1587.645 | 1598.874 |
|  |  | sigma | 0.7949 | 0.2634 | 0.4152 | 1.5218 |  |  |
|  |  | Q | 1.1504 | 0.5612 | 0.0504 | 2.2504 |  |  |
| **Chemotherapy** | Exponential | rate | 0.0121 | 0.0013 | 0.0097 | 0.0150 | 912.03 | 915.067 |
|  | Weibull | shape | 1.2353 | 0.1182 | 1.0241 | 1.4900 | **909.555** | **915.629** |
|  |  | scale | 0.0047 | 0.0023 | 0.0018 | 0.0123 |  |  |
|  | Gamma | shape | 1.3121 | 0.1722 | 1.0145 | 1.6969 | 909.998 | 916.072 |
|  |  | rate | 0.0175 | 0.0035 | 0.0119 | 0.0259 |  |  |
|  | Lognormal | meanlog | 4.0529 | 0.1235 | 3.8109 | 4.2949 | 920.361 | 926.435 |
|  |  | sdlog | 1.2922 | 0.1062 | 1.0999 | 1.5182 |  |  |
|  | Gompertz | shape | 0.0099 | 0.0048 | 0.0005 | 0.0194 | 909.915 | 915.989 |
|  |  | rate | 0.0088 | 0.0017 | 0.0059 | 0.0130 |  |  |
|  | Log-logistic | shape | 1.4792 | 0.1399 | 1.2289 | 1.7804 | 912.429 | 918.503 |
|  |  | scale | 56.9126 | 5.8941 | 46.4574 | 69.7207 |  |  |
|  | Generalized gamma | mu | 4.3869 | 0.1324 | 4.1273 | 4.6465 | 911.316 | 920.427 |
|  |  | sigma | 0.7280 | 0.1861 | 0.4410 | 1.2016 |  |  |
|  |  | Q | 1.2127 | 0.4759 | 0.2799 | 2.1455 |  |  |
| **Results of PFS** | | | | | | | | |
| **Cemiplimab plus chemotherapy** | Exponential | rate | 0.0185 | 0.0013 | 0.0162 | 0.0212 | 2096.733 | 2100.476 |
|  | Weibull | shape | 1.2330 | 0.0716 | 1.1003 | 1.3816 | **2076.608** | **2084.094** |
|  |  | scale | 0.0076 | 0.0022 | 0.0043 | 0.0133 |  |  |
|  | Gamma | shape | 1.4208 | 0.1226 | 1.1998 | 1.6825 | 2083.453 | 2090.939 |
|  |  | rate | 0.0287 | 0.0034 | 0.0227 | 0.0362 |  |  |
|  | Lognormal | meanlog | 3.5842 | 0.0664 | 3.4540 | 3.7144 | 2079.1 | 2086.586 |
|  |  | sdlog | 1.0779 | 0.0556 | 0.9743 | 1.1925 |  |  |
|  | Gompertz | shape | 0.0054 | 0.0034 | -0.0013 | 0.0121 | 2096.33 | 2103.816 |
|  |  | rate | 0.0161 | 0.0019 | 0.0128 | 0.0202 |  |  |
|  | Log-logistic | shape | 1.6305 | 0.0946 | 1.4552 | 1.8268 | 2086.862 | 2094.348 |
|  |  | scale | 36.1494 | 2.2697 | 31.9638 | 40.8832 |  |  |
|  | Generalized gamma | mu | 3.7014 | 0.1078 | 3.4901 | 3.9127 | 2079.425 | 2090.654 |
|  |  | sigma | 1.0070 | 0.0784 | 0.8645 | 1.1729 |  |  |
|  |  | Q | 0.2894 | 0.2220 | -0.1456 | 0.7245 |  |  |
| **Chemotherapy** | Exponential | rate | 0.0321 | 0.0029 | 0.0269 | 0.0383 | 1111.725 | 1114.762 |
|  | Weibull | shape | 1.3439 | 0.0930 | 1.1733 | 1.5392 | **1085.734** | **1091.808** |
|  |  | scale | 0.0095 | 0.0033 | 0.0048 | 0.0188 |  |  |
|  | Gamma | shape | 1.7340 | 0.1985 | 1.3856 | 2.1700 | 1093.538 | 1099.612 |
|  |  | rate | 0.0591 | 0.0083 | 0.0448 | 0.0779 |  |  |
|  | Lognormal | meanlog | 3.0889 | 0.0759 | 2.9401 | 3.2377 | 1095.733 | 1101.807 |
|  |  | sdlog | 0.9044 | 0.0584 | 0.7968 | 1.0266 |  |  |
|  | Gompertz | shape | 0.0085 | 0.0047 | -0.0008 | 0.0178 | 1110.709 | 1116.783 |
|  |  | rate | 0.0269 | 0.0037 | 0.0205 | 0.0353 |  |  |
|  | Log-logistic | shape | 2.0487 | 0.1542 | 1.7677 | 2.3744 | 1098.109 | 1104.183 |
|  |  | scale | 22.3154 | 1.5426 | 19.4878 | 25.5533 |  |  |
|  | Generalized gamma | mu | 3.2562 | 0.1029 | 3.0545 | 3.4579 | 1092.867 | 1101.978 |
|  |  | sigma | 0.8255 | 0.0638 | 0.7094 | 0.9605 |  |  |
|  |  | Q | 0.4333 | 0.1986 | 0.0440 | 0.8225 |  |  |

**Supplementary Table 2.** Associated Costs and Disutility of Grade ≥ 3 Treatment-Related Adverse Events

| **Adverse Event^a^** | **No. of patients (%)** | **Costs in 2021 USD^c^** | **Reference** | **Disutility** | **Reference** |
| --- | --- | --- | --- | --- | --- |
| **Cemiplimab plus chemotherapy^b^** | | | | | |
| Fatigue, asthenia | 13 (4.17) | 10549.09 | Konidaris et al, 2020 | 0.2880 | Nafees et al, 2017 |
| Decreased appetite, nausea, vomiting | 3 (0.96) | 17405.97 | Wong et al, 2018 | 0.2040 | Nafees et al, 2017 |
| Anaemia | 31 (9.94) | 8150.39 | Konidaris et al, 2020 | 0.0720 | Freeman et al, 2015 |
| Diarrhoea | 4 (1.28) | 17005.30 | Wong et al, 2018 | 0.2160 | Nafees et al, 2017 |
| Neutropenia | 18 (5.77) | 18774.14 | Wong et al, 2018 | 0.3480 | Nafees et al, 2017 |
| Thrombocytopenia | 8 (2.56) | 4,934 | Jeong et al, 2021 | 0.003 | Jeong 2021 et al. |
| Weighted averaged |  | 2844 |  | 0.044 |  |
| **Chemotherapy^b^** | | | | | |
| Fatigue, asthenia | 3 (0.96) | 10549.09 | Konidaris et al, 2020 | 0.2880 | Nafees et al, 2017 |
| Decreased appetite, nausea, vomiting | 0 (0) | 17405.97 | Wong et al, 2018 | 0.2040 | Nafees et al, 2017 |
| Anaemia | 10 (3.21) | 8150.39 | Konidaris et al, 2020 | 0.0720 | Freeman et al, 2015 |
| Diarrhoea | 0 (0) | 17005.30 | Wong et al, 2018 | 0.2160 | Nafees et al, 2017 |
| Neutropenia | 9 (2.88) | 18774.14 | Wong et al, 2018 | 0.3480 | Nafees et al, 2017 |
| Thrombocytopenia | 2 (0.64) | 4,934 | Jeong et al, 2021 | 0.003 | Jeong 2021 et al. |
| Weighted averaged |  | 936 |  | 0.015 |  |

^a^Our analysis only included and evaluated grade ≥ 3 treatment-related adverse events.

^b^Number within treatment arm: cemiplimab plus chemotherapy (N = 312), chemotherapy (N = 153).

^c^Calculated as an average cost of toxicity using the weighted frequency of occurrence. This value was used in the base-case model.

**Reference**

Konidaris et al, 2020 Assessing the Value of Cemiplimab for Adults With Advanced Cutaneous Squamous Cell Carcinoma: A Cost-Effectiveness Analysis. doi:10.1016/j.jval.2020.09.014

Wong et al, 2018 Assessment of costs associated with adverse events in patients with cancer. DOI: 10.1371/journal.pone.0196007

Nafees et al, 2017 Nafees B, Lloyd AJ, Dewilde S, Rajan N, LorenzoM. Health state utilities in non–small cell lung cancer: an international study. Asia Pac J Clin Oncol. 2017;13(5):e195-e203. doi:10.1111/ajco.12477

Freeman et al, 2015 Freeman K, Connock M, Cummins E, et al. Fluorouracil plasma monitoring: systematic review and economic evaluation of the My5-FU assay for guiding dose adjustment in patients receiving fluorouracil chemotherapy by continuous infusion. Health Technol Assess. 2015;19(91):1-321. doi:10.3310/hta19910

Jeong E, Wang C, Wilson L, et al. Cost-effectiveness of adding ribociclib to endocrine therapy for patients with HR-positive, HER2-negative advanced breast cancer among premenopausal or perimenopausal women. Front Oncol. 2021; 11:658054. doi: 10.3389/fonc.2021.658054
